# Time and frequency domain analysis of heart rate variability in cattle affected by bovine spongiform encephalopathy

**DOI:** 10.1186/1756-0500-4-259

**Published:** 2011-07-25

**Authors:** Timm Konold, Gemma E Bone, Marion M Simmons

**Affiliations:** 1 Pathology & Host Susceptibility, Neuropathology, Animal Health and Veterinary Laboratories Agency Weybridge, New Haw, Addlestone, KT15 3NB, UK

## Abstract

**Background:**

Heart rate variability (HRV) analysis is a method to assess the function of the autonomic nervous system. Brainstem nuclei that influence HRV are affected by vacuolar changes and accumulation of disease-associated prion protein (PrP^d^) in bovine spongiform encephalopathy (BSE) resulting in clinical signs suggestive of an increased parasympathetic tone. It was hypothesised that BSE in cattle causes changes in the autonomic nervous system; this was tested by comparing HRV indices derived from 1048 electrocardiograms, which were recorded from 51 naturally or experimentally infected cattle with BSE confirmed by postmortem tests, 321 clinical suspect cases or cattle inoculated with potentially infectious tissue without disease confirmation and 78 BSE-free control cattle.

**Findings:**

Statistically significant differences were found for low or high frequency power, their normalised values and ratio when the last recording prior to cull or repeated recordings were compared but only between male and female cattle of the three groups and not between groups of the same gender, even though BSE cases of each gender appeared to be more nervous during the recording. The same findings were made for heart rate, deviation from the mean RR interval and vasovagal tonus index when repeated recordings were compared. BSE cases with severe vacuolar changes in the parasympathetic nucleus of the vagus nerve had a significantly lower low:high frequency power ratio but not a lower heart rate than BSE cases with mild vacuolation, whereas severity of vacuolar changes in the solitary tract nucleus or intensity of PrP^d ^accumulation in both nuclei did not appear to have any affect on either index. Abnormalities in the electrocardiogram were detected in 3% of the recordings irrespective of the BSE status; sinus arrhythmia was present in 93% of the remaining recordings.

**Conclusions:**

HRV analysis was not useful to distinguish BSE-positive from BSE-negative cattle grouped by gender, and HRV indices appeared to be mainly influenced by gender. There is agreement with earlier studies that vacuolar changes in the brainstem may be associated with an increased parasympathetic tone in BSE and that abnormalities in an electrocardiogram can be detected in cattle without evidence of heart disease.

## Background

Studies on the pathogenesis of classical bovine spongiform encephalopathy (BSE) in cattle have indicated that spread of the infectious agent from the intestine to the central nervous system occurs via the autonomic nervous system [1-3]. Vacuolation and accumulation of disease-associated prion protein (PrP^d^) is most evident in nuclei of the brainstem at the level of the obex, particularly the solitary tract nucleus and the parasympathetic (dorsal motor) nucleus of the vagus nerve [4,5] and this pathological phenotype appears to be conserved, even in cattle experimentally infected with BSE [6,7]. The axons of neurons from both nuclei contribute to the formation of the motor component of the vagus nerve [8]. An increased vagal tone, resulting in bradycardia, has been reported in cattle with BSE, presumably caused by functional changes in parasympathetic brainstem nuclei [9,10]. Analysis of fluctuation in the heart rate provides the opportunity for a non-invasive tool to assess autonomic control of the heart [11], which has been used in various species, including cattle [12]. This method has shown that oral challenge of cattle with the BSE agent resulted in changes in heart rate variability (HRV), and it was suggested that it may provide a tool to develop a live animal test for BSE [13].

The present study aimed to investigate the hypothesis that alterations in the autonomic nervous system were detectable in confirmed BSE cases and different to those in cattle reported as BSE suspects or experimentally inoculated cattle unconfirmed by postmortem tests and unchallenged or saline-inoculated control cattle.

### Animals

All procedures involving animals were carried out in accordance with the Animal (Scientific Procedures) Act 1986, under licence from the United Kingdom Government Home Office, which was granted following an internal ethical review process within the Veterinary Laboratories Agency (VLA).

Examinations were carried out on 450 adult cattle, which belonged to various studies conducted at the VLA Weybridge and comprised 366 castrated male, referred to as 'male', and 84 female cattle (see Table 1). The vast majority (428 cattle, 95%) were pure- or crossbred Holstein-Friesians that were found in all studies except for study 6; other breeds were Aberdeen Angus crossed with Danish dairy breeds in study 6 (ten) and pure- or crossbred Simmental (one), Brown Swiss (one), Belgian Blue (one), Charolais (two), Limousin (three) and Hereford (four animals) in study 7. Prior to the recording of the electrocardiogram (ECG) all animals were also subjected to a neurological examination to determine the clinical BSE status [14].

**Table 1:** Number of examined animals and their recordings per study

Study	BSE-positive cattle ^a^ N animals N recordings (female/male)	BSE-negative cattle ^b^ N animals N recordings (female/male)	Controls ^c^ N animals N recordings (female/male)
1	2 (0/2) 2 (0/2)	220 (0/220) 244 (0/244)	9 (0/9) 9 (0/9)

2	9 (5/4) 12 (8/4)	27 (8/19) 38 (18/20)	19 (9/10) 27 (17/10)

3	14 (0/14) 38 (0/38)	36 (0/36) 101 (0/101)	8 (0/8) 28 (0/28)

4	2 (0/2) 12 (0/12)	1 (0/1) 27 (0/27)	11 (0/11) 80 (0/80)

5	2 (0/2) 23 (0/23)	10 (0/10) 100 (0/100)	12 (0/12) 238 (0/238)

6	7 (2/5) 9 (2/7)	0	2 (1/1) 2 (1/1)

7	15 (15/0) 15 (15/0)	27 (27/0) 29 (29/0)	17 (18/0) 18 (17/0)

**Total**	**51 (22/29)** **111 (25/86)**	**321 (35/286)** **536 (46/490)**	**78 (27/51)** **401 (36/365)**

^a ^Cattle with BSE confirmed by postmortem tests.

^b ^BSE field suspects or cattle inoculated with tissue/body fluids from naturally infected BSE cases or from BSE/scrapie-challenged animals, which were not confirmed as BSE cases by postmortem tests.

^c ^Cattle inoculated with saline solution or BSE/scrapie-free tissue or not challenged.

1 Cattle intracerebrally inoculated with various tissues collected from cattle orally dosed with classical BSE brainstem homogenate and saline-inoculated controls [45,46].

2 Cattle orally dosed with 1 g or 100 g classical BSE brainstem homogenate and culled at various times post challenge and undosed controls [1].

3 Cattle orally dosed with 1 g-1 mg classical BSE brainstem homogenate and undosed controls [7].

4 Cattle orally dosed with 100 g classical BSE brainstem homogenate and undosed controls as well as controls orally dosed with 100 g BSE-free brainstem homogenate sourced from New Zealand.

5 Cattle intracerebrally inoculated with scrapie brain homogenate, saline-inoculated controls and controls inoculated with scrapie-free brain [6] as well as cattle orally inoculated with 100 g scrapie brain homogenate and undosed controls.

6 Cattle intracerebrally inoculated with atypical BSE brain homogenate (L-type or H-type BSE) as well as unchallenged controls.

7 BSE suspects reported by the farmer and healthy controls purchased from farms [14].

### Heart rate monitoring

In total, 1048 ECGs were recorded using the base-apex lead with disposable skin-adhesive electrodes (Unilect, Unomedical Ltd. Stonehouse, UK) and added gel (Lectron II, Pharmaceutical Innovations, Newark, USA) to improve conductivity. The negative electrode was placed at the caudal angle of the left scapula, the positive electrode placed at the left intercostal space caudal to the olecranon and the ground electrode placed at the left paralumbar fossa. Recordings were made whilst the animal was restrained in a crush after a neurological examination had been conducted and usually lasted for at least 310 seconds. Recordings were amplified, digitised and processed using computer software Spike2 (version 4, CED, Cambridge, UK). Each R-peak of a QRS complex was marked. The HRV was determined from tachygrams of instantaneous heart rate, which were produced by plotting the length of the time between successive R peaks of a 5-minute ECG segment against cumulative time. Fast Fourier transform was performed on each tachygram, which separates the heart rate signal into its frequency components and quantifies them in terms of their relative intensity as power [15], using 1024 points to calculate the power spectrum. The upper limit for the low frequency (LF) power band, representative of sympathetic and parasympathetic activity, was 0.16 Hz, the upper limit for the high frequency (HF) power band, representative of parasympathetic activity at the respiratory frequency, was 0.7 Hz. This is a slight modification from the settings recommended recently [12], which suggested an upper limit of 0.58 Hz for adult cattle, equivalent of a maximum respiratory rate of 35 per min. As the respiratory rate of cattle monitored during the ECG recording could be as high as 42 per min, which was also reported in a study on the breathing frequency in ruminants [16], the upper limit was increased to 0.7 Hz. HF and LF power were expressed as absolute values and in normalised units (proportion of LF or HF power contributing to the total power) [12]. In addition, the LF:HF power ratio was determined as a measure of sympathovagal balance. The calculation of these values was not possible for 62 ECG recordings from 14 cattle where the available ECG segment was less than 310 seconds (e.g. QRS complexes could not be identified correctly over a longer period). However, all recordings were used to determine the mean heart rate (HR), the mean R-R interval, the deviation of the mean R-R interval (in %) as a measure of sinus arrhythmia and the vasovagal tonus index (VVTI, natural logarithm of the variance in the RR-intervals) [17], based on a recording with 72 QRS complexes, which was the number of QRS complexes identified in the shortest continuous ECG. A variation of the R-R intervals of more than 10% was interpreted as sinus arrhythmia [18]. Any animal with a mean HR of less than 44 beats per minute (bpm) was considered to display bradycardia and any animal with a HR above 120 bpm was considered to display tachycardia [19].

Table 1 lists the number of animals with recorded ECGs grouped by study and BSE status and the total number of recordings within each group.

The behaviour during the ECG recording was assessed for 1041 recordings from 447 cattle and scored using a three point scale: 0 = quiet, 1 = restless (e.g. shifting weight, kicking, occasional or frequent tail swishing or head shaking in the absence of flies), 2 = myoclonus (tremor or sudden startle responses), although for analytical purposes 1 and 2 were combined since both scores may indicate an increased level of nervousness.

### Disease confirmation and pathology

The final BSE status was determined by postmortem testing (histopathology, immunohistochemistry, Western immunoblot) [20] of a section of the brainstem at the level of the obex and up to seven additional sections rostral to the obex according to established methods [21]. For simplification, the term 'BSE status' is used irrespective of the inoculum used for experimental challenges (classical or atypical BSE, scrapie).

Tissues were fixed in 10% formol saline and processed to paraffin wax using standard histological methods. Sections were either stained with haematoxylin and eosin for the assessment of vacuolar lesions, or immunolabelled with the anti-PrP monoclonal antibody R145 at a dilution of 1:400 as described elsewhere [21]. The severity of vacuolar changes in the solitary tract nucleus (STN) and the parasympathetic (dorsal motor) nucleus of the vagus nerve (PNV) was scored semi-quantitatively in BSE-positive cattle on a scale of 0 (no vacuolar changes) to 4 (numerous vacuoles) by a single examiner according to established methods [22]. In addition, the same examiner scored the severity of intraneuronal and - separately - neuropil PrP^d ^immunolabelling in these nuclei on a scale similar to that previously used in sheep scrapie [23] (0 = no immunolabelling; 1 = weak immunolabelling, 2 = moderate immunolabelling, 3 = strong immunolabelling, 4 = very strong immunolabelling) and also determined the immunolabelling pattern (punctuate or stellate). Given the multiple projects contributing animals to the study and the long time period over which animals were studied, it was not possible to standardise absolutely all parameters associated with tissue fixation and processing. However, all animals were killed at the VLA and handled in a consistent way, which minimises potential variation. For the purposes of the semi-quantitative scoring in this study, all obex sections were immunolabelled on the same technical run to enable comparison of the relative extent of immunolabelling with the minimum of technical variation.

## Findings

### Comparison of data and discussion

#### Heart rate variability indices and BSE status

Animals were grouped according to gender within their corresponding challenge group and BSE status: female and male BSE positive challenged/naturally infected cattle, BSE negative challenged/suspect cattle and BSE negative controls.

When all 1048 ECG recordings were considered, 13 (2.9%) of 450 cattle displayed bradycardia (two on two recordings) and five (1.1%) displayed tachycardia, which are detailed in Table 2.

**Table 2:** Cattle with bradycardia and tachycardia

Animal	HR	Gender	BSE status	Study	Animal Status	Age [m] (MPI)	Inoculum
Bradycardia							

D121	31	Female	Positive	2	Inoculated	65 (59)*	100 g BSE brain

CM871	34	Male	Negative	4	Control	71*	None

D133	38	Female	Positive	2	Inoculated	62 (57)*	100 g BSE brain

	42					61 (56)	

CM875	38	Male	Negative	4	Control	71*	None

CV2721	38	Female	Positive	7	BSE field suspect	55*	Natural infection

CV2915	39	Female	Negative	7	BSE field suspect	70*	None

D11	40	Female	Negative	2	Control	95	None

D276	40	Female	Negative	2	Inoculated	94 (89)	1 g BSE brain

D281	41	Female	Negative	2	Inoculated	93 (88)	1 g BSE brain

	43					94 (89)*	

CL679	42	Male	Negative	1	Control	102 (99)*	Saline

CV2758	43	Female	Positive	7	BSE field suspect	105*	Natural infection

Tachycardia							

CM879	121	Male	Negative	4	Control	101*	100 g BSE-free brain

CN1029	131	Male	Negative	1	Inoculated	88 (84)*	Thymus^1^

CM902	131	Male	Negative	3	Control	151*	None

CL705	141	Male	Negative	1	Inoculated	99 (96)*	Kidney^2^

CV2714	153	Female	Negative	7	BSE field suspect	32*	None

* Last (of multiple recordings) or single ECG recording prior to cull.

HR = heart rate, MPI = months post inoculation.

^1 ^Pool from cattle orally challenged with 100 g BSE brainstem and culled at 6 months post challenge.

^2 ^Pool from cattle orally challenged with 100 g BSE brainstem and culled at 18 months post challenge.

**Figure 1:**
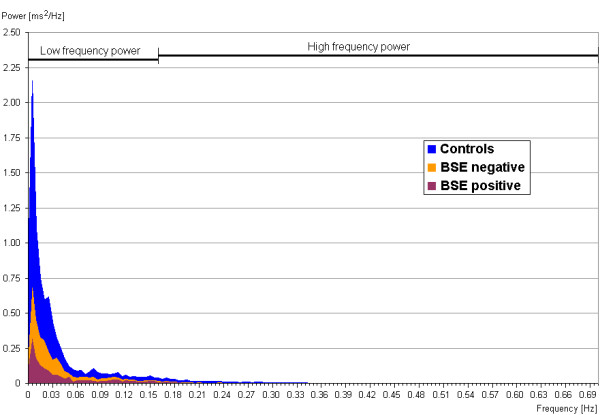
**Median power spectra for control, BSE-negative challenged/suspect and BSE-positive cattle after frequency domain analysis**. The median power spectrum is the grand median of the power spectra from all animals in each group, calculated from the power for each frequency (in 0.005 Hz steps). BSE-negative cattle were cattle experimentally challenged with material from BSE-exposed or BSE-positive cattle or clinical field suspects, which were not confirmed as BSE cases by postmortem tests. BSE-positive cattle were cattle with a postmortem test diagnosis consistent with BSE.

Tables 3 and 4 list the time and frequency domain indices in male and female cattle in the three different groups (controls, BSE-challenged/exposed and postmortem test-negative, BSE-challenged/exposed and postmortem test-positive) based on the last recording prior to cull. An example of the median power spectra for the three female cattle groups is given in Figure 1.

**Table 3:** Frequency domain indices in the different groups based on the last ECG recording

Group ^a^ (N cattle)	LF power [ms^2^] Median (range)	HF power [ms^2^] Median (range)	LF norm Median (range)	HF norm Median (range)	LF:HF power ratio Median (range)
1. Female control (24)	5575.0 (608.2-73164.0)	255.9 (8.6-13019.0)	95.88 (38.49-99.86)	4.12 (0.14-61.51)	23.3 (0.6-697.5)

2. Female negative (35)	2511.0 (117.7-49095.0)	151.0 (0.4-12872.0)	89.76 (49.46-99.92)	10.24 (0.08-50.54)	8.8 (1.0-1198.0)

3. Female positive (19)	2380.0 (394.6-9671.0)	267.9 (3.7-8866.0)	90.73 (35.02-99.36)	9.27 (0.64-64.98)	9.8 (0.5-156.1)

4. Male control (46)	5304.0 (914.2-39518.0)	140.8 (4.1-4037.0)	98.12 (45.44-99.94)	1.88 (0.06-54.56)	52.2 (0.8-837.5)

5. Male negative (270)	5447.0 (154.5-84706.0)	79.6 (3.3-20985.0)	98.58 (46.65-99.97)	1.42 (0.04-53.35)	69.3 (0.9-2856.0)

6. Male positive (27)	6717.0 (1142.0-37547.0)	102.2 (11.3-5229.0)	97.33 (72.72-99.95)	2.68 (0.05-27.28)	36.4 (2.7-1831.0)

***P*-value **(Kruskal-Wallis test) **Significant differences between groups **(Dunn's test)	< 0.0001 2 & 4, 5, 6 (**) 3 & 4, 5, 6 (*)	0.0022 None	< 0.0001 2 & 5 (*) 3 & 4 (*), 5 (***)	< 0.0001 2 & 5 (**) 3 & 4 (*), 5 (***)	< 0.0001 2 & 5 (**) 3 & 4 (**), 5 (***)

^a ^Negative: cattle inoculated with tissue/body fluids from BSE/scrapie-challenged or naturally infected BSE cases as well as reported BSE suspects that were negative for BSE by postmortem tests.

Positive: challenged or naturally infected BSE suspects that were positive for BSE by postmortem tests.

Dunn's post test *P *values are expressed as * (*P *< 0.05), ** (*P *< 0.01) and *** (*P *< 0.001).

**Table 4:** Time domain indices and behaviour scores in the different groups based on the last ECG recording

Group^a^	Heart rate [bpm] Median (range)	Deviation from mean interval [%] Median (range)	VVTI Median (range)	Behaviour scores: N animals^b^
Female control	66 (45-109) N = 26	28.8% (7.8-84.7%) N = 25	10.8 (1.9-24.3) N = 25	0: 17 (65%) 1: 7 (27%) 2: 2 (8%)

Female negative	61 (40-154) N = 36	28.5% (2.9-91.7%) N = 35	8.9 (2.2-33.5) N = 35	0: 20 (59%) 1: 8 (23%) 2: 6 (18%)

Female positive	62 (32-89) N = 22	29.6% (6.7-140.9%) N = 21	9.1 (1.9-28.5) N = 21	0: 5 (23%) 1: 6 (27%) 2: 11 (50%)

Male control	66 (34-132) N = 50	29.8% (10.5-127.2%) N = 48	9.3 (2.5-22.6) N = 48	0: 38 (76%) 1: 11 (22%) 2: 1 (2%)

Male negative	67 (45-142) N = 288	24.8% (6.9-138.8%) N = 277	11.2 (1.5-28.5) N = 277	0: 208 (72%) 1: 57 (20%) 2: 22 (8%)

Male positive	65 (51-108) N = 28	28.1% (7.4-80.1%) N = 27	11.1 (3.2-20.3) N = 27	0: 12 (43%) 1: 4 (14%) 2: 12 (43%)

***P*-value^c^**	ns (0.09)	ns (0.1)	ns (0.1)	Female groups: < 0.0001* Male groups: 0.003*

^a ^Negative: cattle inoculated with tissue/body fluids from BSE/scrapie-challenged or naturally infected BSE cases as well as reported BSE suspects that were negative for BSE by postmortem tests.

Positive: challenged or naturally infected BSE suspects that were positive for BSE by postmortem tests.

^b ^The behaviour score was not assessed in all animals with recorded ECGs.

^c ^Comparison of the heart time domain indices was done by Kruskal-Wallis test (ns = not significant), comparison of the behaviour scores within female and male cattle (score 0 versus scores 1 and 2) was done by Chi-square test.

* Significant differences between BSE-positive cattle and controls as well as BSE-positive and BSE-negative cattle after employing Bonferroni's correction (*P *< 0.03).

Significant differences in any of the indices (*P *< 0.05, Kruskal-Wallis one-way ANOVA [24] and subsequent Dunn's post test [25]; GraphPad Prism, version 5, GraphPad Software, La Jolla, USA) were only found between male and female cattle of the different groups but not within cattle of the same gender (see Table 3). "Additional file 1: graphs" presents the data as box-and-whisker plots for easier comparison.

Regarding the behaviour scores, a significantly higher proportion of female control and BSE-negative cattle was quiet (score 0) during the recording compared to female BSE-positive cattle (*P *= 0.009 and *P *= 0.02 respectively, Chi-Square test with subsequent Bonferroni's *post hoc *test [26]) and - equally - a higher proportion of control and BSE-negative steers were quiet compared to BSE-positive steers (*P *= 0.01 and *P *= 0.003 respectively), whereas there was no significant difference in the proportions between controls and BSE-negative cattle within each gender (see also Table 4).

The analysis of frequency and time domain indices obtained from repeated recordings, whereby the indices for each individual animal were derived from the mean of multiple recordings of this animal (summary measures approach [26]), are displayed in Tables 5 and 6. Significant differences (*P *< 0.05, non-parametric test as above) were again found only between cattle of different gender.

**Table 5:** Frequency domain indices in the different groups based on repeated ECG recordings over time

Group ^a^ (N cattle)	LF power [ms^2^] Median (range)	HF power [ms^2^] Median (range)	LF norm Median (range)	HF norm Median (range)	LF:HF power ratio Median (range)
1. Female control (8)	8604.0 (1668.0-61996.0)	2509.0 (32.3-7844.0)	89.2 (49.92-99.02)	10.8 (0.98-50.08)	13.14 (1.1-170.9)

2. Female negative (9)	6007.0 (1914.0-14428.0)	514.5 (172.2-4588.0)	88.36 (58.65-98.51)	11.64 (1.49-41.36)	10.7 (1.4-74.6)

3. Female positive (1)	2325.0	1076.0	69.37	30.63	6.6

4. Male control (22)	7273.0 (3691.0-45335.0)	110.4 (13.7-596.4)	98.02 (92.52-99.76)	1.98 (0.24-7.5)	125.9 (19.9-814.7)

5. Male negative (45)	6545.0 (2027.0-26832.0)	91.6 (3.3-651.7)	98.95 (90.85-99.83)	1.05 (0.17-9.15)	222.9 18.0-1282.0)

6. Male positive (14)	6960.0 (2486.0-33200.0)	163.6 (18.6-1743.0)	97.86 (86.61-99.84)	2.14 (0.16-13.39)	95.8 (25.3-997.1)

***P*-value **(Kruskal-Wallis test) **Significant differences between groups **(Dunn's test)	ns (0.6)	< 0.0001 1 & 5 (**) 2 & 5 (**)	< 0.0001 1 & 5 (**) 2 & 5 (**)	< 0.0001 1 & 5 (**) 2 & 5 (**)	< 0.0001 1 & 5 (**) 2 & 4 (*), 5 (***)

^a ^Negative: cattle inoculated with tissue/body fluids from BSE/scrapie-challenged or naturally infected BSE cases as well as reported BSE suspects that were negative for BSE by postmortem tests.

Positive: challenged or naturally infected BSE suspects that were positive for BSE by postmortem tests. BSE-positive female cattle were included for completeness, even though the total number was too low for any statistical comparison.

Dunn's post test *P *values are expressed as * (*P *< 0.05), ** (*P *< 0.01) and *** (*P *< 0.001).

**Table 6:** Time domain indices in the different groups based on repeated ECG recordings over time

**Group**^a^	Mean heart rate [bpm] Median (range)	Deviation from mean interval [%] Median (range)	VVTI Median (range)
1. Female control	60 (45-104) N = 9	42.6% (10.6%-74.3%) N = 8	10.8 (5.1-13.4) N = 8

2. Female negative	50 (42-64) N = 9	48.1% (30.7%-76.5%) N = 9	6.4 (3.9-9.9) N = 9

3. Female positive	56 (43-70) N = 2	62.1% (40.9%-83.3%) N = 2	7.4 (6.2-8.7) N = 2

4. Male control	66 (57-106) N = 22	26.3% (13.1%-44.9%) N = 22	11.4 (7.6-19.4) N = 22

5. Male negative	73 (49-114) N = 51	27.2% (8.3%-53.1%) N = 47	13.4 (5.4-28.0) N = 47

6. Male positive	66 (58-94) N = 16	29.0% (14.1%-57.6%) N = 15	13.0 (7.0-22.1) N = 15


***P*-value **(Kruskal-Wallis test) **Significant differences between groups **(Dunn's test)	< 0.0001 2 & 4 (*), 5 (***)	0.0005 2 & 4 (*), 5 (**)	< 0.0001 2 & 4 (*), 5 (***), 6 (**)

^a ^Negative: cattle inoculated with tissue/body fluids from BSE/scrapie-challenged or naturally infected BSE cases as well as reported BSE suspects that were negative for BSE by postmortem tests.

Positive: challenged or naturally infected BSE suspects that were positive for BSE by postmortem tests. BSE-positive female cattle were included for completeness, even though the total number was too low for any statistical comparison.

N = Number of examined cattle

Dunn's post test *P *values are expressed as * (*P *< 0.05), ** (*P *< 0.01) and *** (*P *< 0.001).

Measurement of HRV provides a tool to assess the autonomic nervous system where high frequency variation in heart rate is mediated by the parasympathetic nervous system and low frequency variation is modified by both sympathetic and parasympathetic components [11]. The results presented here do not indicate that HRV analysis is useful as a diagnostic test for BSE in cattle. Admittedly, our study included cattle inoculated by different routes (oral and intracerebral) and with different strains (classical BSE, atypical BSE, classical scrapie), but as all pathologically confirmed cases presented with PrP^d ^accumulation in the brain including at least the STN in the obex it was assumed that all should present with some HRV changes compared to cattle without PrP^d^. However, significant differences were not found between female or male BSE cases and BSE-negative or control cattle of the same gender. In addition, there was a considerable overlap in the values across all groups. A study in calves suggested that LF and HF power and their ratio were indeed unreliable markers of sympathovagal balance because of high individual variation [27]. Similar results were obtained in the present study with the time domain index VVTI, which has been determined in studies of dogs and is easily calculated [17,28].

HRV analysis employed in cattle research has generally only compared cattle of the same gender [29-32]. The fact that significant differences were found for individual HRV indices between the same groups (e.g. LF norm, HF norm and LF:HF power ratio) was not surprising since the parameters are correlated with each other [31]. In humans, time and frequency domain indices are influenced by gender, which also appears to apply to cattle, but also by age [33]. All cattle were adult in the present study (range 18-171 months of age when the last ECG was recorded), and the median age at the last ECG recording of BSE-positive cattle was significantly lower than that of controls or BSE-negative exposed or field suspect cattle (70, 96 and 92 months respectively, *P *< 0.0001 by Kruskal-Wallis test with Dunn's *post hoc *test, data not shown). However, there did not appear to be great correlation between age and HRV indices, such as heart rate, VVTI and LF:HF power ratio, with less than 5% of the variance of each index explained by the relationship with age (Pearson's correlation coefficient *r *< 0.042, data not shown). This is supported by the finding that HRV indices are not significantly different in cattle with advanced age [32].

Surprisingly, there was no evidence of an increased parasympathetic tone in BSE cases compared to BSE-negative cases of the same gender. In an earlier study, the finding of bradycardia in BSE cases, which are easily excitable so that a higher heart rate is expected [34], was associated with an increased parasympathetic tone as a result of a possible excitation or disinhibition of neurons in the brainstem affected by spongiform changes [9,10]. However, in the present study bradycardia was rare and independent of BSE status, the heart rates were not significantly lower in cattle with BSE compared to the other groups and the LF:HF power ratio, which represents sympathovagal balance, was equally not significantly different (lower) although the behaviour score during recording was significantly higher in BSE-positive cattle, i.e. more displayed nervous or restless behaviour and startle reactions or shivering, which should have resulted in an increased sympathetic tone, an increased heart rate and a higher LF:HF power ratio. If the BSE agent caused both sympathetic and parasympathetic nervous dysfunction, HRV analysis would not necessarily detect abnormalities because both systems counteract each other. Increased blood pressure as observed in BSE cases [35] is in fact an indicator of reduced parasympathetic nervous activity because hypertension in human patients has been associated with reduced vagal function [36,37]. In general, cardiovascular control, which influences HRV indices, is very complex, and control mechanisms include higher centres of the brain (hypothalamus, cortex) and the renin-angiotensin system [12], which were not evaluated in the present study.

It has been hypothesised that functional changes may occur in BSE-challenged cattle, which may be detectable by HRV analysis, even though the postmortem diagnosis is negative [13]. In this earlier study, which included some of the cattle we also examined in the present study, perturbation of HRV could be detected at 29-43 months after oral challenge with 1 g BSE brainstem homogenate, but only a few of these cattle were confirmed as BSE cases when culled later [1]. If a test was able to diagnose BSE earlier than the currently used diagnostic postmortem tests, their use as gold standard would be questionable in the absence of any supporting confirmatory data. However, differences in HRV indices between challenged BSE-negative cattle and controls were also only found between male and female cattle and not in groups of the same gender, which suggests that HRV is influenced by gender, with a higher parasympathetic tone (higher HF norm and lower LF:HF power ratio) found in female cattle. In the above mentioned study [13], the authors did not compare HRV indices by gender and used different power spectrum ranges, which may explain the discrepancy between their results and ours.

#### Heart rate variability indices and vacuolation or PrP^d ^accumulation

To assess the effect on neuropathological changes (severity of vacuolation, intensity of PrP^d ^accumulation) on heart rate and sympathovagal balance, groups with a difference of at least two score points were compared (e.g. cattle with vacuolation score 0-1 versus cattle with vacuolation score 3-4 whilst ignoring cattle with score 2; single scores were not compared due to the small sample sizes). This was based on the assumption that due to the subjective assessment only cases in which the scores are two points apart should be considered different from one another.

**Figure 2:**
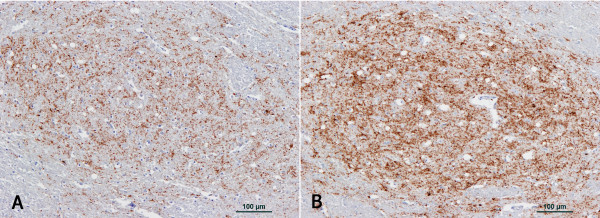
**PrP^d ^immunolabelling in the solitary tract nucleus of two orally infected BSE cases**. **A, B: **Solitary tract nucleus of two BSE cases orally challenged with 100 g (steer CM873) and 10 mg (steer CM923) respectively of BSE brainstem homogenate and culled one day after the last ECG recording. PrP^d ^immunolabelling with antibody R145. Neuropil PrP^d ^immunolabelling intensity is weaker in A (score 2, CM923, heart rate 57 beats per minute) compared to B (score 4, CM873, heart rate 108 beats per minute).

There was no significant difference in the heart rate between BSE-positive cattle with mild neuropathological changes and those with severe changes in the PNV and the STN (*P *> 0.05, Mann-Whitney test, GraphPad Prism) although there was a tendency for BSE cases with severe vacuolar changes in the PNV to have a lower heart rate than those with mild vacuolation. Figure 2 gives an example of two BSE cases where more intense PrP^d ^immunolabelling was found in the BSE case with a considerably higher heart rate. The LF:HF power ratio was significantly lower in BSE cases with a high vacuolation score than those with a low score in the PNV (see Table 7 and additional file 1: graphs giving a graphical representation of the values).

**Table 7:** Association of heart rate and sympathovagal balance with neuropathological changes in selected nuclei of the obex

	Neuroanatomical area	Score	N cattle	Heart rate [bpm] Median (range)	*P*-value	LF:HF power ratio Median (range)	*P*-value
Vacuolation	Parasympathetic nucleus of the vagus nerve	0	13	59 (54-86)	ns	31.5 (5.3-164.8)	0.01
score	(neuropil*)	≥ 2	9	46 (32-76)	(0.08)	5.1 (0.5-37.5)	
	
	Solitary tract nucleus	0-1	12	61 (54-89)	ns	33.1 (5.3-87.6)	ns
	(neuropil*)	3-4	21	64 (32-84)	(0.99)	10.2 (0.5-1831.0)	(0.15)

PrP^d^	Parasympathetic nucleus of the vagus nerve	0	10	69 (54-81)	ns	43.0 (2.7-164.8)	ns
intensity	(intraneuronal)	2	17	65 (39-109)	(0.90)	24.6 (1.1-1831.0)	(0.56)
	
score	Parasympathetic nucleus of the vagus nerve	0-1	11	66 (58-89)	ns	27.5 (6.7-146.0)	ns
	(neuropil)	3-4	26	63 (32-109)	(0.13)	16.5 (0.5-1831.0)	(0.34)
	
	Solitary tract nucleus	0-1	6	66 (55-81)	ns	27.1 (2.7-164.8)	ns
	(neuropil*)	3-4	33	63 (32-109)	(0.63)	12.3 (0.5-1831.0)	(0.65)

* Intraneuronal vacuolation in the PNV was too infrequent to be used for comparison; vacuolation and PrP^d ^accumulation in the STN was exclusively in the neuropil.

ns = not significant (*P *> 0.05).

The type of immunolabelling in the PNV [punctuate only (11 cattle) versus stellate and punctuate immunolabelling (31 cattle) in the neuropil] did not appear to have any effect on the values measured since the differences were not significant (*P *> 0.05, Mann-Whitney test, GraphPad Prism).

The findings did not suggest that the intensity (or type) of PrP^d ^accumulation in the PNV and STN has any measurable effect on the parasympathetic nervous system, particularly an increase in the parasympathetic tone. We have previously also found a poor association between PrP^d ^immunolabelling in selected neuroanatomical areas in the brain and clinical signs in goats with scrapie [38]. In contrast, the sympathovagal balance was indeed affected by more severe vacuolar changes in the PNV: it resulted in a significantly lower LF:HF power ratio suggestive of an increase in the parasympathetic tone, which is in agreement with earlier studies [9,10]. Vacuolation *per se *is not believed to cause functional deficits in BSE of cattle but is associated with a more severe clinical disease [39] and the finding in the present study may merely indicate that an increase in vacuolation is more likely to cause deregulation of the autonomic nervous system although increased vacuolation in the STN specifically did not have a significant effect.

#### Other abnormalities

**Figure 3:**
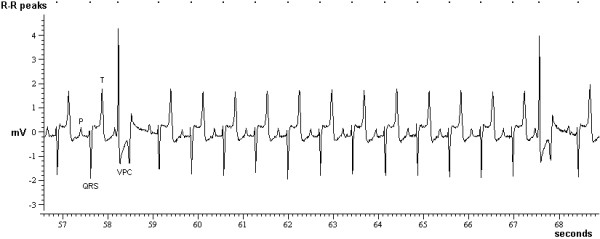
**Ventricular premature complex in a BSE case**. ECG of a 91 month-old Holstein-Friesian cow (CV2720), which presents with occasional abnormally shaped QRS-T complexes (ventricular premature complex: VPC). Mean heart rate 75 beats per minute.

**Figure 4:**
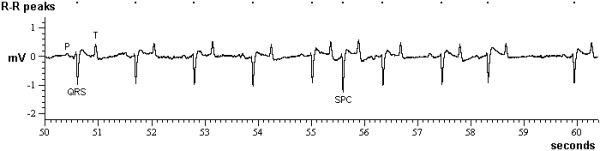
**Supraventricular premature complex in an inoculated, BSE-negative steer**. ECG of an 89 month-old Holstein-Friesian steer (CN1090) intracerebrally inoculated at five months of age with spleen homogenate collected from cattle orally dosed with BSE brainstem homogenate and culled at 10 months post inoculation. A normally shaped QRS complex occurs prematurely (supraventricular premature complex: SPC) and is not preceded by a P wave. There is also sinus arrhythmia present. Mean heart rate 57 beats per minute.

**Figure 5:**
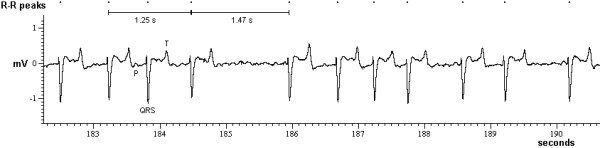
**Atrial fibrillation in a BSE case**. ECG of a 55 month-old cow (D114) orally dosed at 6 months of age with 100 g BSE brainstem homogenate. The QRS complexes are normally shaped but the R-R intervals are completely irregular, sometimes greater than two previous intervals, with irregular movements of the baseline (flutter waves). Mean heart rate 83 beats per minute. The ECG appeared normal with regular R-R intervals 46 days earlier.

Abnormal electrical activity of the heart was identified in 32 (3%) recordings from 21 (5%) cattle, which consisted of ventricular premature complexes in five [one BSE-positive cow (see Figure 3), four BSE-negative, inoculated steers], supraventricular premature complexes in 12 cattle [one BSE-positive steer, nine BSE-negative, inoculated steers (see Figure 4) and two control steers] and atrial fibrillation in four cattle [one BSE-positive cow (see Figure 5), one BSE-negative, inoculated cow and one male and female control]. Eleven of these cattle had repeated recordings, and with the exception of a BSE-positive and a control steer, the abnormalities were only found in single ECG recordings, five of which were the last recordings prior to cull. Sinus arrhythmia was present in the remaining 972 (93%) ECG recordings from 424 (94%) cattle.

There was no clinical evidence of cardiac disease in these cattle and gross abnormalities of the heart were not found. Cardiac dysrhythmias can indeed occur in clinically healthy cattle [40] and may also disappear spontaneously [41], which is in agreement with the present study where abnormalities were not consistently detected on repeated ECG recordings. Atrial fibrillation and its paroxysmal form can also occur in clinically apparently healthy cattle and may be caused by increased sympathetic or parasympathetic tone [42]. Thus, it is impossible to conclude that they are caused by the accumulation of the BSE agent in the autonomic nervous system if found in BSE-positive cattle, particularly since abnormalities were detected in cattle of all groups regardless of BSE test or inoculation status.

Cardiac arrhythmia, which includes sinus arrhythmia, can be detected in up to 30% of horses [43] but is usually not detected in cattle except on very careful clinical examination or examination of an ECG [19]. A study of ECGs from 952 cattle has found sinus arrhythmia in 9% of cases [40]. The authors did not provide a definition of their classification but when we used an interbeat variation of 10% as criteria [18] it was surprising to find that this occurred in 93% of our cases, excluding those with other abnormalities in the electrical activity of the heart. Only ECGs without artefacts - after individual checking and manual editing if necessary - were analysed so that the correct R-R intervals could be determined; HRV analysis was not done in cattle with evident ECG abnormalities, such as atrial fibrillation or premature complexes, and frequency domain indices were only calculated from a continuous 5-minute ECG recording. This is within the recommended standard for HRV analysis, which suggests using short-term recordings that are free from ectopic beats, missing data and noise [44]. It is possible that the digital recording of ECGs and subsequent analysis of R-R intervals is more accurate than the measurements of R-R intervals on ECG paper and thus identified more cases with sinus arrhythmia. BSE did not appear to have an effect on the display of sinus arrhythmia because the proportion of BSE-positive, BSE-negative exposed or clinical suspect and control cattle within each gender that displayed sinus arrhythmia at the last recording prior to cull was similar (data not shown).

## Conclusions

If grouped by gender HRV analysis was unable to distinguish cattle with BSE from cattle with a negative postmortem test result. Differences in HRV indices appeared to be mainly influenced by gender. More severe vacuolar changes in the brainstem may lead to an increase in the parasympathetic tone in BSE, which is in agreement with previous studies. This study confirms that cattle may have ECG abnormalities without evidence of heart disease.

## Competing interests

The authors declare that they have no competing interests.

## Authors' contributions

TK and GEB recorded the electrocardiograms and assessed the animal's behaviour. TK carried out the analysis and drafted the manuscript. MMS was responsible for the neuropathological examination and performed the lesion scoring. All authors read and approved the final manuscript.

## Supplementary Material

Additional file 1 **Additional file 1 - Graphs**. Box-and-whisker plots of HRV indices determined from the last recording prior to cull and repeated recordings and grouped by gender and BSE/inoculation status (BSE positive, BSE negative, control male and female). Box-and-whisker plots of selected HRV indices grouped by neuropathological changes in the PNV and STN in the brainstem. Click here for file
