# Supplementary material for: Time and frequency domain analysis of heart rate variability in cattle affected by bovine spongiform encephalopathy
Source: BMC Res Notes. 2011 Jul 25;4:259. doi: 10.1186/1756-0500-4-259 (PMC3169472; doi:10.1186/1756-0500-4-259)
Supplement: Additional file 1 — Graphs. Box-and-whisker plots of HRV indices determined from the last recording prior to cull and repeated recordings and grouped by gender and BSE/inoculation status (BSE positive, BSE negative, control male and female). Box-and-whisker plots of selected HRV indices grouped by neuropathological changes in the PNV and STN in the brainstem. [file 1756-0500-4-259-S1.DOC]

Comparison of frequency and time domain indices between groups

### 1. Last recordings prior to cull


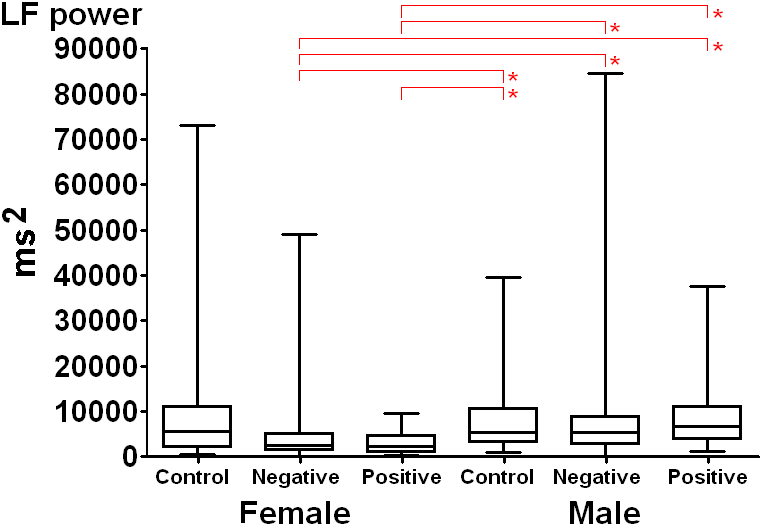


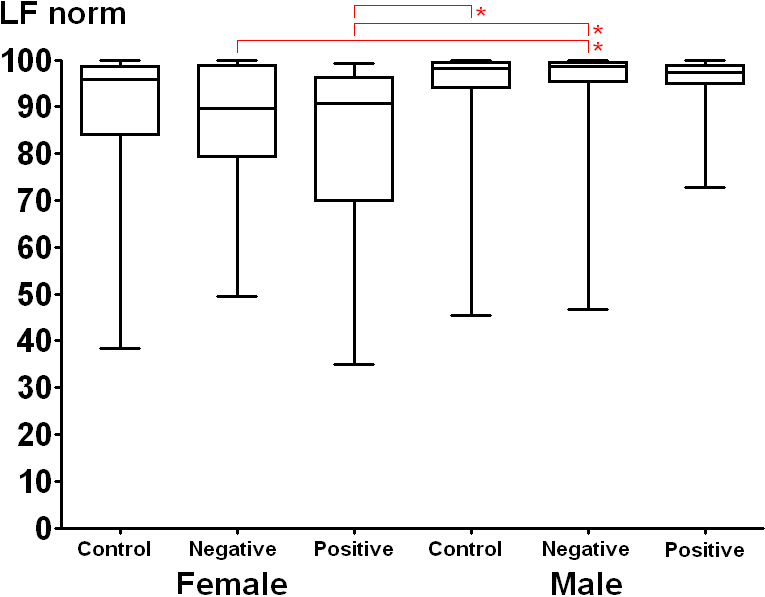


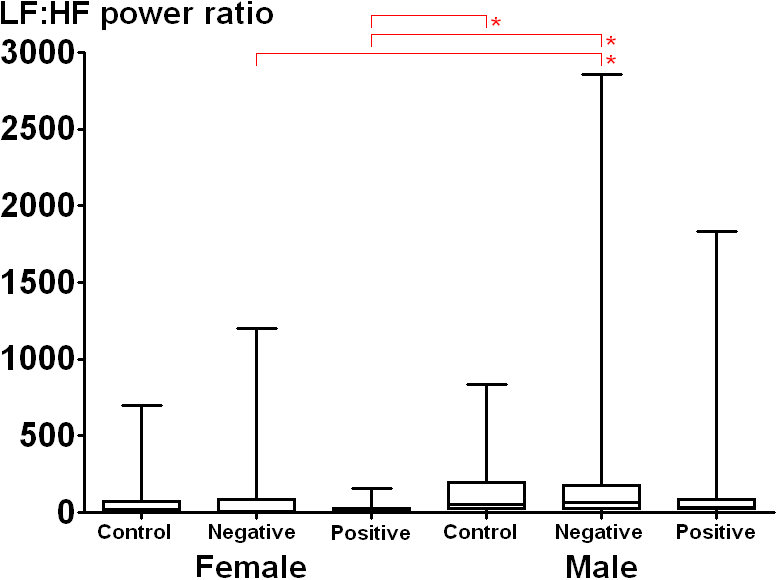


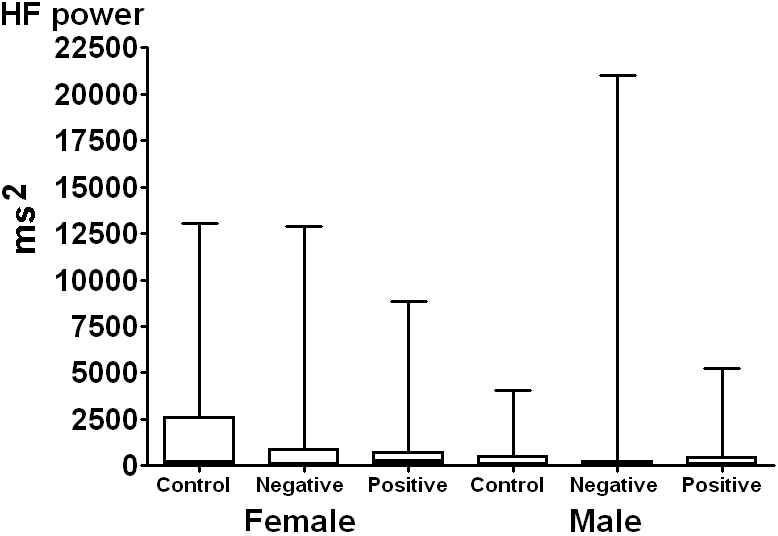


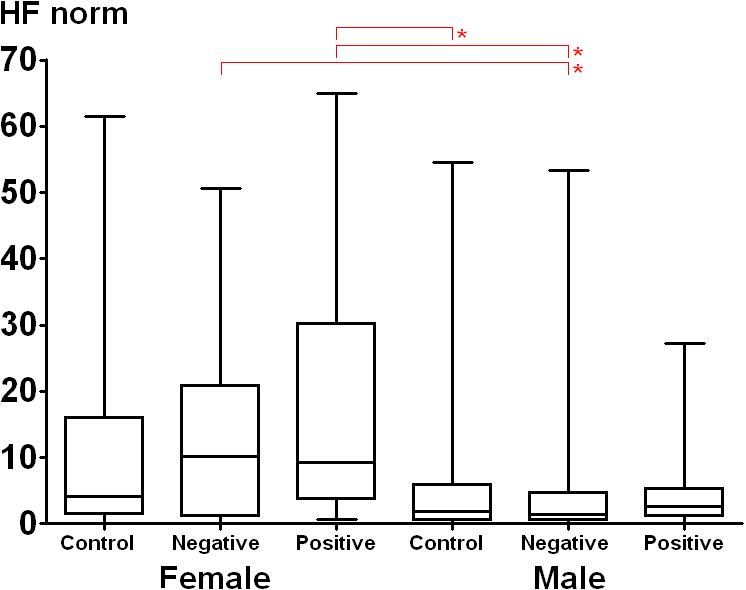


Plots show the minimum, maximum and median.

* Group values that were significantly different (*P*<0.05) are indicated by red lines.


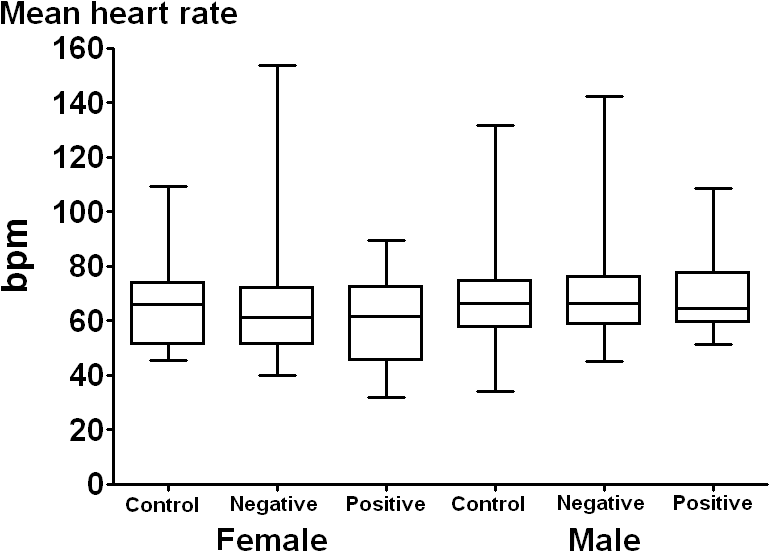


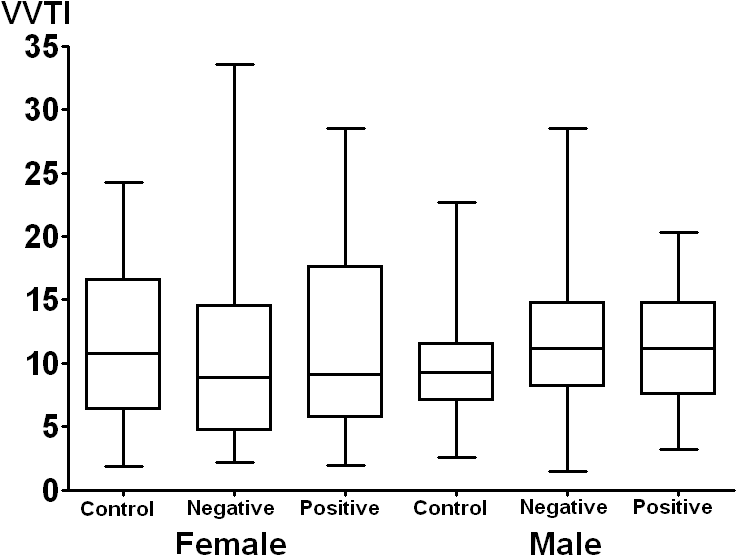


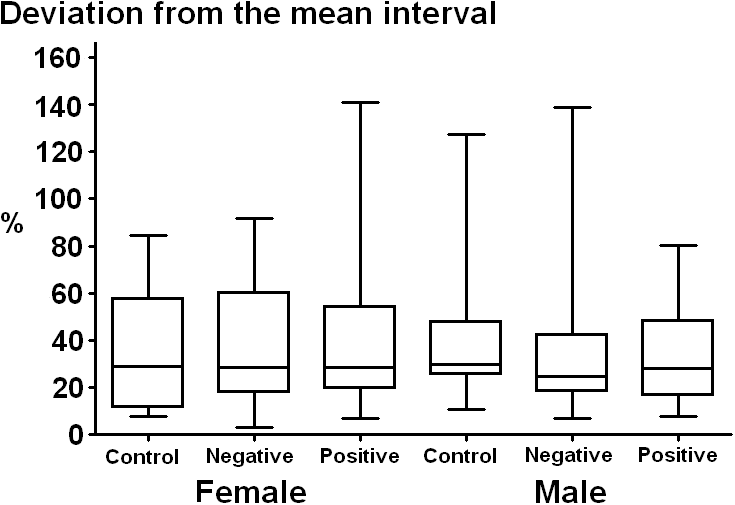


Plots show the minimum, maximum and median.

### 2. Repeated recordings (mean values)


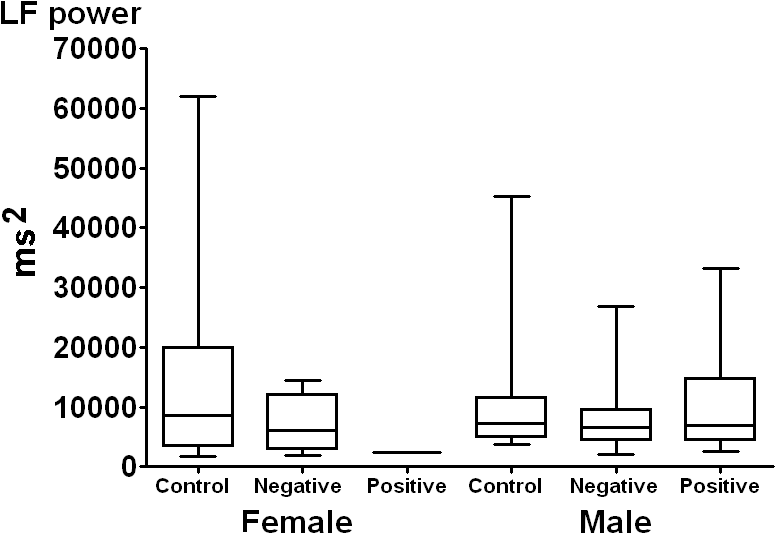


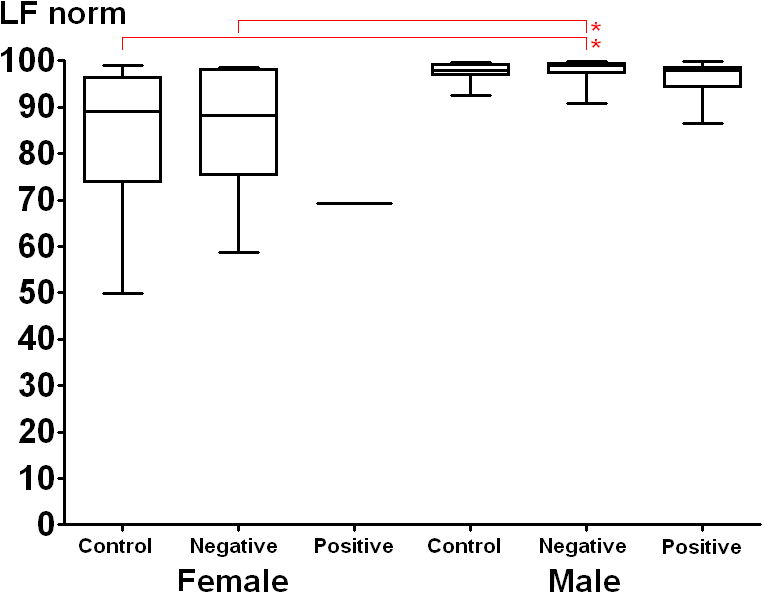


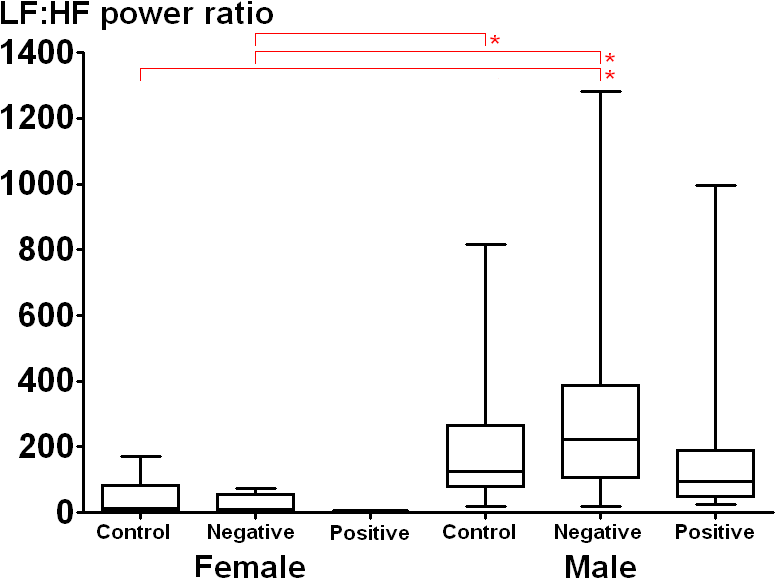


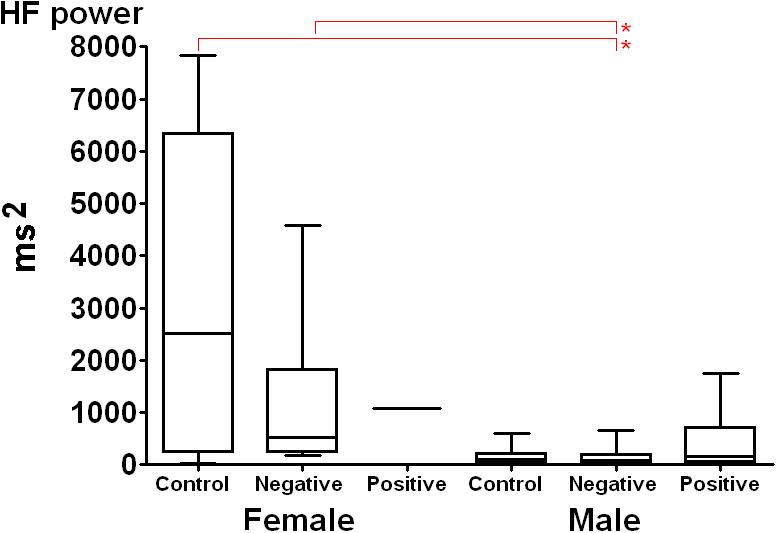


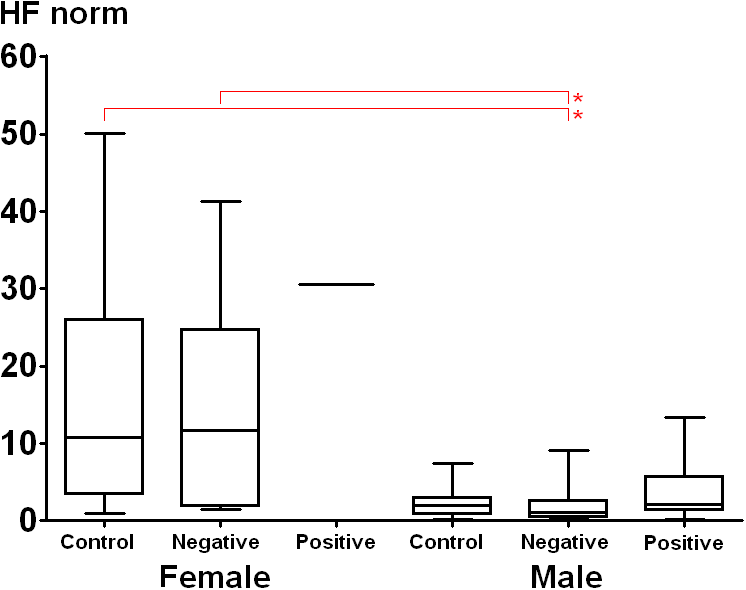


Plots show the minimum, maximum and median.

* Group values that were significantly different (*P*<0.05) are indicated by red lines.


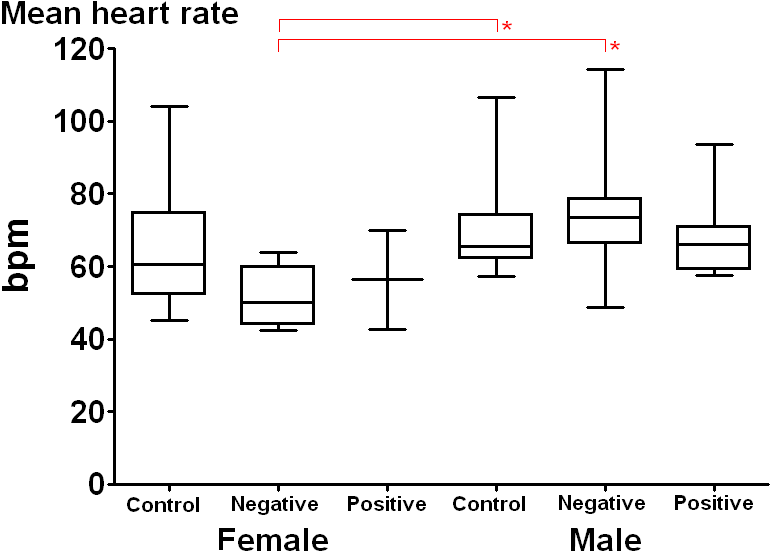


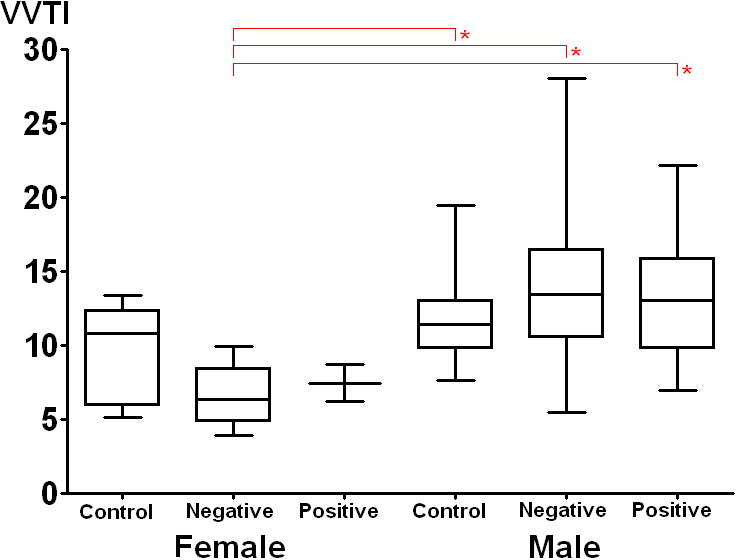


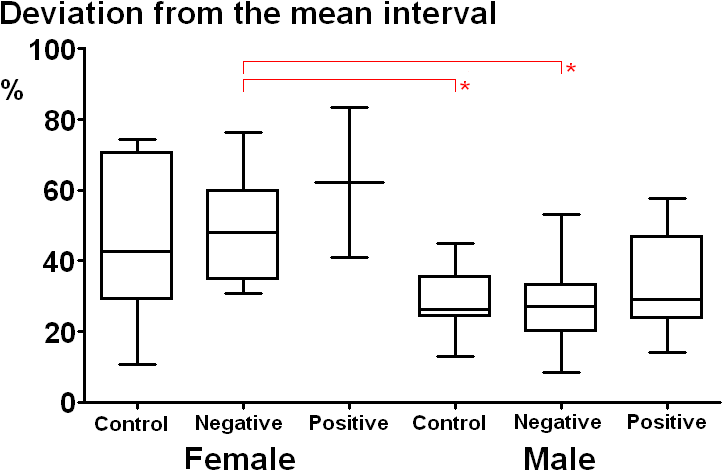


Plots show the minimum, maximum and median.

* Group values that were significantly different (*P*<0.05) are indicated by red lines.

Association between selected time and frequency domain indices and neuropathological changes

### 1. Heart rate


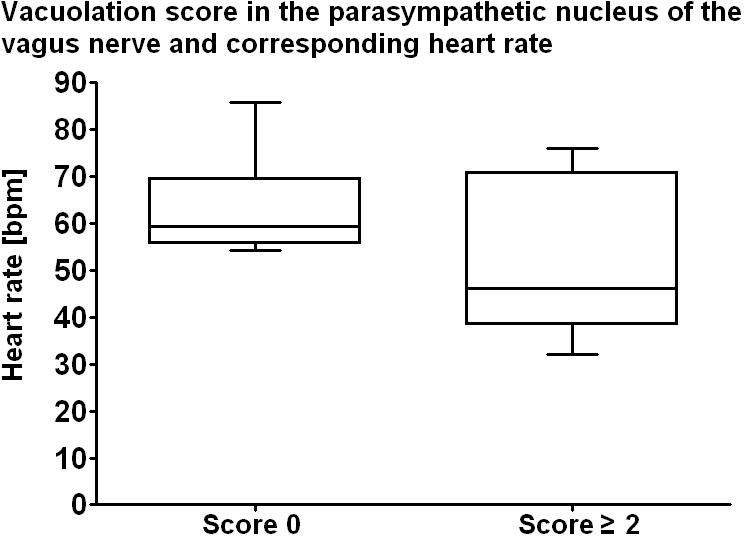


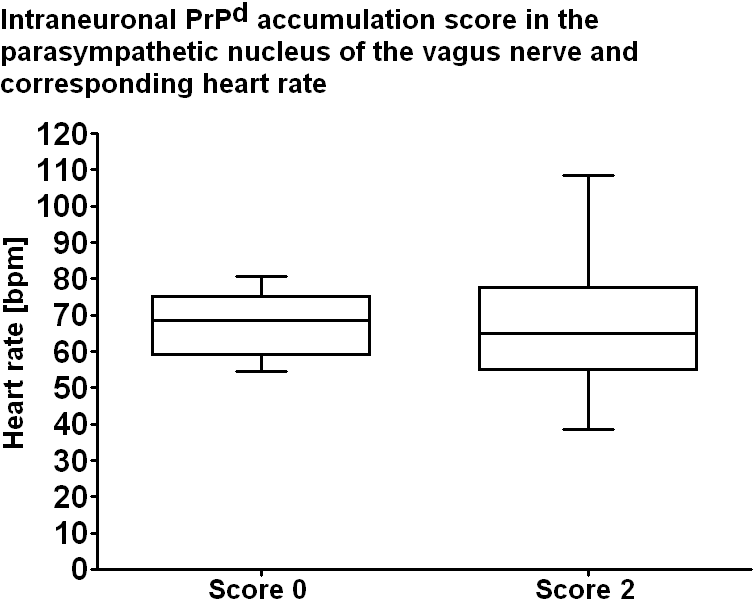


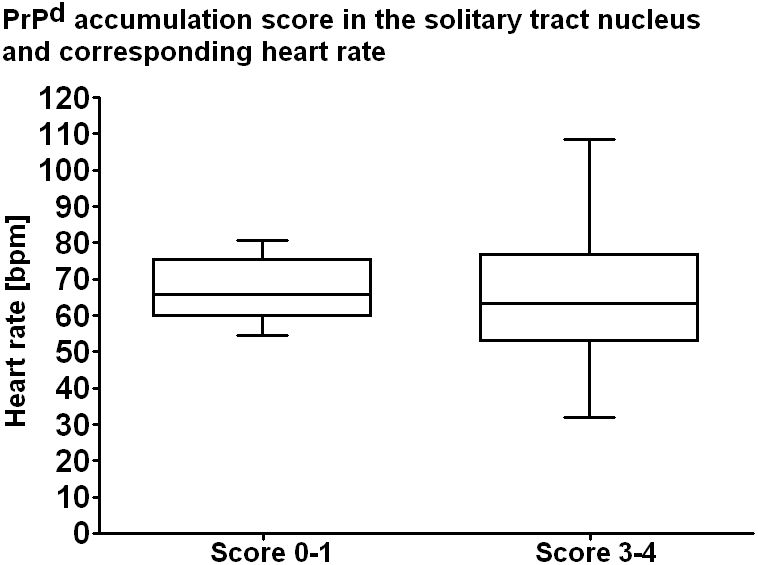


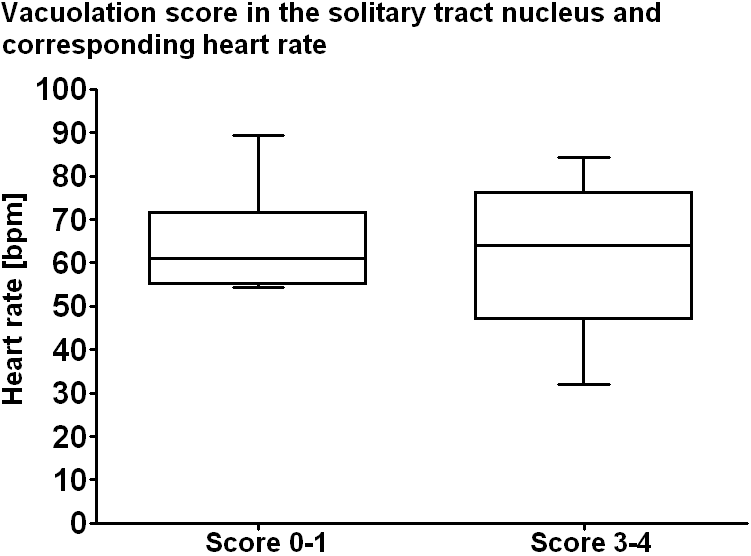


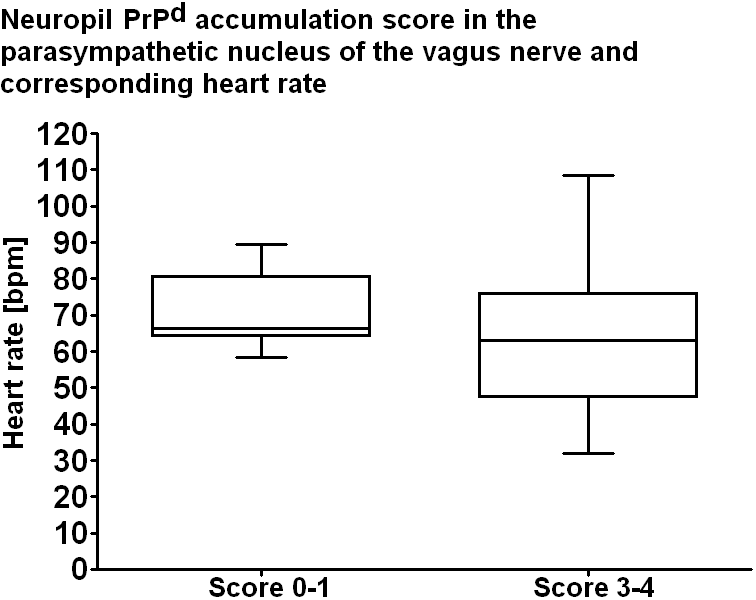


Plots show the minimum, maximum and median.

### 2. LF:HF power ratio (sympathovagal balance)


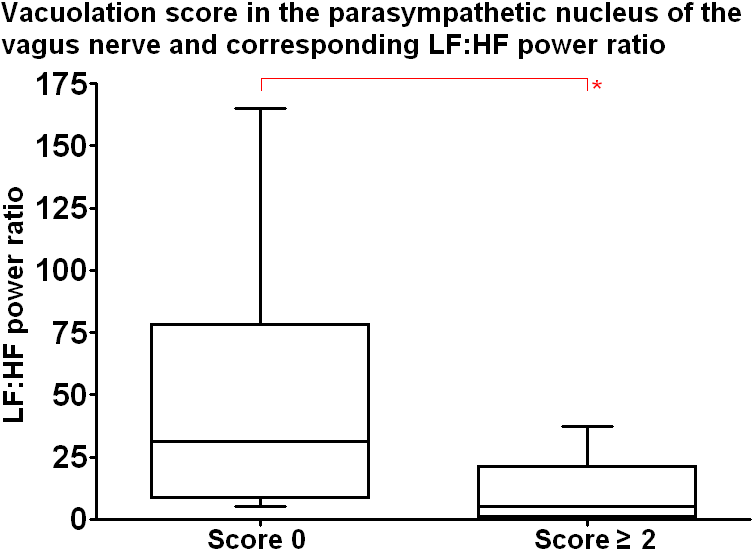


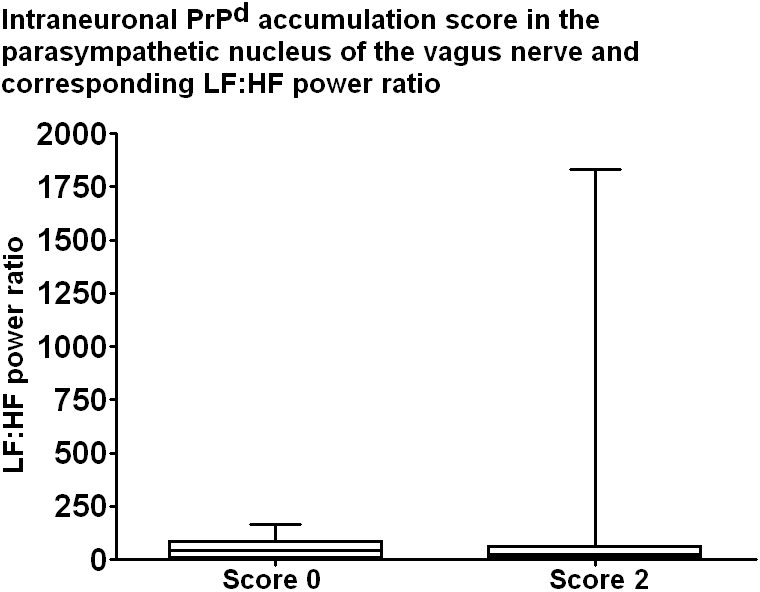


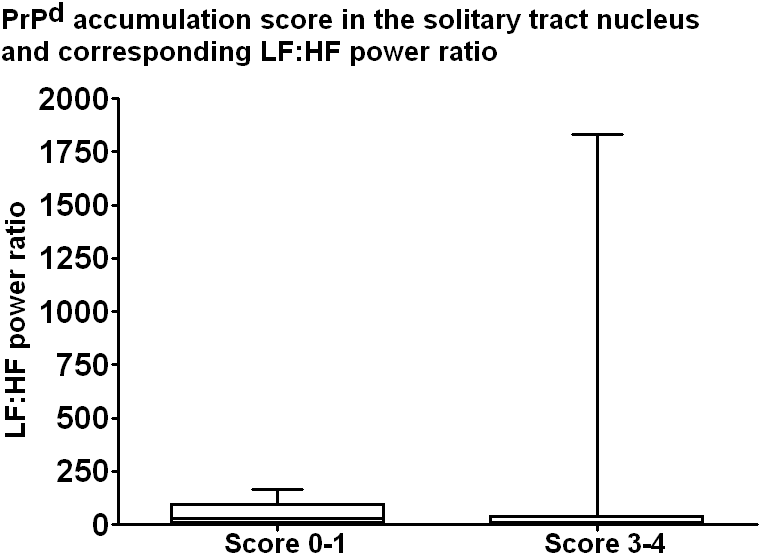


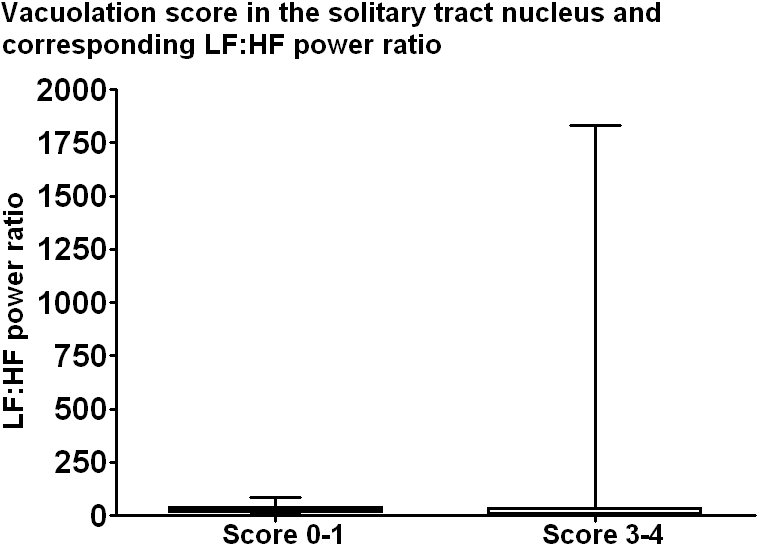


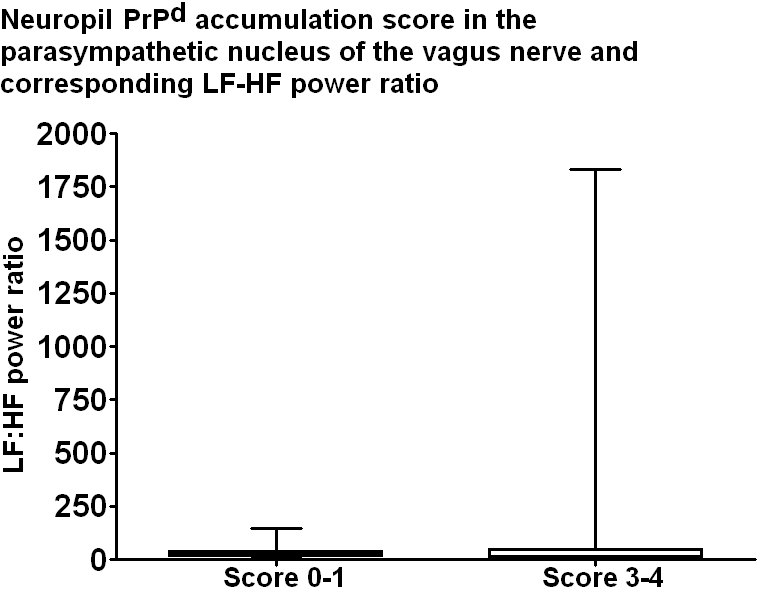


Plots show the minimum, maximum and median.

* Group values that were significantly different (*P*<0.05) are indicated by red lines.
